# Conceptualization and practices in digital health: voices from Africa

**DOI:** 10.4314/ahs.v22i1.77

**Published:** 2022-03

**Authors:** Gertjan van Stam

**Affiliations:** Masvingo Zimbabwe

**Keywords:** Digital health, Africa, ethnography

## Abstract

This paper presents voices from Africa on digital health in Africa. These voices were gleaned during interviews and an online, focus group session in May 2020, during which 30 experts across Africa, among others from the South, were asked about their experiences and observations on the conceptualisation of, and practices in, digital health in their respective communities and countries. Extensive input was provided, both orally and textually. The quotes gathered and presented in this paper indicate that there is a distinct need for the respectful co-development of digital health interventions in Africa. In addition, the quotes show how a one-size-fits-all solution approach does not exist, it is not a solution to Africa. Further, the community-focus, fit, and fragmentation of existing activities digital health interventions is questioned. The narratives provide a rich resource indicating capable and local agency and the need to address power-differences in international health development.

## Introduction

Digital health (or eHealth, derived from electronic health) is defined as “the cost-effective and secure use ofInformation and Communications Technologies ICT in support of health and health-related fields, including healthcare services, health surveillance, health literature, and health education, knowledge and research”^1^. The World Health Organisation argues digital health is essential for universal health coverage, a prime focus of global health^2^. Global health operates in a “multifaceted and dynamic global context characterized by great diversity among societies in norms, values, and interests, as well as by large inequalities in the distribution of health risks and the resources to address them”^3^,^2^. Digital health, usestilising digital platforms to host digital health services and provides tools for digital health interventions ideally that serve medical experts, clients, health systems and others through the contextual and sound use of information and communication technologies (ICT) and their derivatives in computer and data science, such as, for instance, through machine learning and artificial intelligence (AI).

The global growth of digital connectivity, devices, and platforms has a direct effect on the available channels for communication and information. The impacts of ICTs are pervasive in all aspects of life, including healthcare and the prevention of disease. These impacts are facilitated through new opportunities for information exchange between health clients and health (care) providers. Digital health, therefore, represents an emerging field that should be conceptualised not as subservient to current forms of health care, but as an integral part of such care^43^. It lends scalability and ability to additional forms of health care and, thus, overlays and coexists with them. Advanced information technology and the integration of information systems through telecommunication networks and services, including Internet applications has the potential to increase operational efficiency, support decision-making processes, and enhance management effectiveness^54^.

Digital health, like all health interventions, needs input from social, cultural, health, and information systems. It creates both opportunities and threats for contemporary health management – as it affects the central features of health systems. Digital ealth challenges the reach of (local) health institutes and the role of national health governance. ICTs, through their global nature, alter or bridge spatial imaginaries^65^. Therefore, digital health affects established practices in both public and private health management and healthcare systems.

Although feats of engineering are crucial to create and sustain digital health, it is not only about technical developments. Incorporating ICTs in support of health and health-related fields is a state-of-mind – a way of thinking and an attitude. It needs commitment to networked thinking aimed at improving the quality of health care, locally, nationally and globally. However, the common digital health narratives appear to be Eurocentric, with little diversity in the voices expressed 7–9. In this paper, I focus on registering and mainstreaming voices from Africa.

## Method

This ethnographic paper presents ‘voices from Africa’. It is an extension of years of applied research in the South and, more specifically, during interactions probing key stakeholders from Asia, Africa, and Latin America in May 2020. Thirty invitations went out to a convenience sample of high-level experts. Among these were directors of African IT companies, directors of African community networks, directors of digital health infrastructures at ministries of health in African countries, directors of various innovation hubs in Africa, and major players in te African health scene. The invitations were sent to actors with a record of articulating Southern perspectives in digitisation at national and international gatherings, both written and oral. Two-thirds of the invitees responded, which response were enhanced through led to five in depth written responses and five structured, in depth interviews by means of a direct connection over Skype. These interviews were performed, recorded and transcribed by the author.

On 25 May 2020, the results of the interviews were presented and discussed and expanded upon during a twohour, interactive focus group session held via Zoom with representatives from Africa, Asia, and Latin America and interactions with observers in Europe, framed by an inspirational compilation of quotes (available at http://tiny.cc/mms-dh).

## Results

### An African narrative

In an effort to harvest critical inputs, all respondents were asked to reflect on the current challenges and future needs in digital health, and an early draft of a transnational framework for digital health being produced by Medicus Mundi Switzerland (subsequently published in March 2021106), and other frameworks guiding digital health interventions they were aware of.

In this section, I present the African voices, verbatim, from their observations and contributions, in a form that reads like a narrative. Each paragraph is a separate quote from a different author/speaker. Where oral, these quotes were recorded, transcribed and lightly edited. Original recordings and transcriptions are held by the author.^1^

### On methodology

*There is need for a methodological approach that focuses on finding a common groundto provide a starting point for co-creation. In the classical African context, it is called ‘putting heads together’. This means it is not hierarchical, andll ideas and views count and matter. It is about collective knowledge building and sharing, in which diversity of views as a strength is respected, and the questions of equal participation, ownership, trust, commitment and co-creation are inherently addressed. Such mechanisms are based on experience and skills, expertise and deep knowledge of the subject which is orally shared* (Oratio).

*Such an approach must be anchored in the philosophy of humanness. This only works when there is a shift in the mindset to value other lives as we value ours. This philosophy speaks to the idea of the universalism of humanness. It is only through such lenses that there can be true realization that there is no lesser being, regardless of geographical location. The history of European-African relations teaches that the framing process, as influenced by ideological (spiritual, political or cultural, and mercantilist/economic) imperatives, informs the treatment of others...*

*... there is a need for a value set, decentering, sharing, of togetherness, of collaboration atthe right level. Not like the former concepts of collaboration where the North brings things to the South and the South executes them under the North's direction*.

*It is rarely the case that Euro-American/Western based ways of conducting any intervention in the South are questioned and get a strong recommendation towards considering the field feedback provided* (or to be provided) by the locals in the South.

*There is a great need to have every possible voice represented. That is one of the greatest ways of making sure that eHealth works in a setting like ours. Because it is not the same setting as in the West. Things do not work the same. There is really no copy and pasting that you can do when it comes to Southern Africa. Everything is completely different.*

### On the integrity of digital health

*Recent developments in digital health point to challenges and opportunities as technology intersects with context specific socio-cultural, economic and political dynamics. One critical area in relation to the implementation of digital health technology in Africa is culture. This relates to the intersection between culture and the emergence and use of digital technologies. Secondly, there is an increase in innovative products out of local creativity and entrepreneurship. These innovations are home grown, culturally and technologically adapted to the local conditions. They are cost effective, readily accessible and sustainable. These are products of frugal innovation, something that Europe will need to pay attention to once it has gone through a mental shift (accepting the reality that other epistemologies and ontologies do exist and are both valid and reliable or simply put, ‘our way is not the only way’). These innovations point to the future prospect of technological leapfrogging in Africa and its role as a skills base and market for advanced products*.

There were many things that people didn't want to say, because, generally, we say things to be diplomatic, but we do not face problems head-on.

*The health sector attracts a lot of donors. The tendency has been mainly siloed data systems, siloed implementation. In most cases, these are not really interoperable, with even national systems.As a result, the health sector struggles with a lot of siloed implementations, which is mainly driven by donor funded initiatives*.

The issue of ethics is very, very important. ... *You would find when a researcher comes, for instance, he would come from somewhere (let's say the West) and come and collect the data, extract that data. As a local person, you provide all the information because of trust – you trust people, and you try and provide every information* ... *And what happens, from our experience, when that researcher gets back to his or her university, they will use that data without the knowledge of the local people. And that is a very big problem.*

*There is a need to address the pain encapsulated in the relationship between Africa and Europe.*

*It is about respecting each other, each other's culture, respecting each other's values, respecting each other's geographical differences.*

*If I want to implement a digital health initiative in a certain area, I first of all have go to the stakeholders in those areas and engage them, so they can tell you what they want. We have that problem in many places, where the technology is deemed to be owned by the technology provider.*

*It is not because you have implemented it inone African country that you can implement it inanother African country. It is not possible.*

*We talk about it, but inan African country for instance, as in many other countries, I haven't yet seen a single digital service that I think is useful... I have not seen any digital health service that is there and is working for the people.*

*We do not take any opportunity to contest, to say ‘we do not need it’. We need to have the courage to say: ‘we do not need it, we need something else’.*

*Sometimes, the donor has good intentions, he comes with something that he thinks is a good idea, that he wants to transfer from one country to another country, or he wants to transfer technology, but we do not have the courage to say ‘it cannot work, we do not need it’. We need to talk together. This will already eliminate 80% of the projects, and if we eliminate 80% of what I call ‘noise’, then we will hear things that can work – if we talk together, if you go there and ask people what they really want*.

### On community engagement

*Equity comes into play, if we adopt digital health – or any technology for that matter – we have to scan the landscape, look at the communities: what do they want and what do they lack for them to be digital? Ignorance comes into play when we come in with technologies and ignore the cultures of the areaswe ignore the configuration of the communities... this will result in technologies not really being embraced by communities*.

*If we go in with the mindset that ‘no, they do not have anything’, that is where we got it wrong for the last fifty years*.

*In digital health, relationships should first be built. The most important thing... is first to be in a relationship*.

*You cannot engage the communities through paperwork. First you have to visit. Do exchange visits, and rural-to-rural visits.*

*Written information is difficult to interpret because we are an oral community. It needs to be interpreted inindigenous languages... We can only transmit the information via the spoken word.*

*[Digital health must involve the community to participate – that means engaging the community, which includes the thought leadership (the local talent). That is decentering, where both the donor and also the recipient community is participating – where it is co-developed. Also, where there are some ethics. Ethics is about respecting each other, being honest, being transparent.*

*[There is a need to focus on the traditional structures, from the headman to the chief. Because, the majority of the population in Zambia are from rural areas and these are politically governed by traditional leaders. Traditional leaders should be aware of what is happening, they should be informed using different platforms, not only the Internet, but also through community radio stations.*

*The community has to identify the challenges or problems that affect them. And then, the donor, for example, should be able to listen to the problems identified by the community. Any aid that is given to that particular community should be in line with the needs of the community, what the community has identified, not what has been identifiedby others.*

*You get a lot of knowledge by interacting with local people. That knowledge does not belong to you, it belongs to the people.*

### On workforce enhancement

*Who went to the rural areas and asked them ‘what are your problems’? Did you go there and ask them is this your issue, actually?*

*We need to ask people how to help them help themselves.*

*In our country, 70% to 80% of people are illiterate. And I look at mobile health, mHealth services and so on, and projects saying we are going to use SMS, going to do this, going to do that... How? How are they going to do this? Then we have fancy things, can have smartphones and apps... How? Who has got a smartphone and to plug it where?*

*[There is a need for the consolidation of all the digital health systems because they continue to be developed in a fragmented way every day.... Accept that local capacity can be groomed to spearhead development in the South.*

*We need to make sure that it is in phase with the realities on the ground.*

*We need voice, audio and video. That is it. Talk to people, repeatedly - and that's it.*

*Digital health is a technical thing. It is not like other development scenarios... It requires expertise in health, and it requires expertise in digital*.

*We are devising nice ideas that we cannot implement, because the underlying infrastructure is not there.*

*Technology should be used as the first check-box. Missingthe infrastructure, there is no point trying to make a discourse about this.... Solve the issue or move on to another problem.*

*In digital health, technology should be at the front. We need to first look into the possibility of doing this, from a technical point of view, before we start arguing about what this will bring and so on. Can we do it even?*

### On thought leadership

*Projects must not come in, but must come out of the community.*

### Development should be community driven.

*Train leaders locally. These leaders should be trained by their fellow local people*.

Sovereignty gives our data a belonging, it gives our stories the authority to say: ‘*according to the community inlocation they think that digital health is ..., or they see that digital health is ...*’ *That is the sovereignty aspect that you need and that you require*.

*I fear that when you come... to the ministry and say: ‘We have money for this project and that project’, I am pretty sure that people are not looking at the outcome of the project. They are looking at the process of the project, what they will gain personally, the travels they will make, the kind of incentives they will have and not about the final goal of the project.*

*Local leadership is about embodied knowledge; what you have learnt from the time you were born and what you have observed*.

## Discussion

New manifestations of health provisioning, in particular, ‘the trans-nationalisation of the local’ through ICTs for digital health interventions, raises important issues of engagement, as can be deduced from the quotes above. Globalised services emerge that compete with those provided by local health institutes. They are ushered in with ‘free money’ from donors, set up as islands with their own ways of addressing issues like data management. In many parts of Africa, such developments are in the early stage of conceptualisation and review. At present, it is estimated that less than a quarter of the population in Africa are using facilities providethrough the Internet; hence, digital health still has a long way to go, beyond the stage of sensitisation, testing, amending and small-scale implementation, before it can become ubiquitously available and operational. Evidence as to how digital health can enhance the wellbeing of the disenfranchised, or potentially harm them by changing the health management and care landscape, is still scarce.

In Africa, a variety of initiatives can be categorised as digital health. Many of these are nationwide initiatives, most of them are at the platform level. Here, new threats arise in manners echoing colonialist venturing. An example of such a framing can be recognised in this quote from an MIT Technology Review in 2016:

From a data-production perspective, activities are like lands waiting to be discovered. Whoever gets there first and holds them gets their resources – in this case, their data riches.^117^

This is not the general view from African stand points.

**Figure 1:**
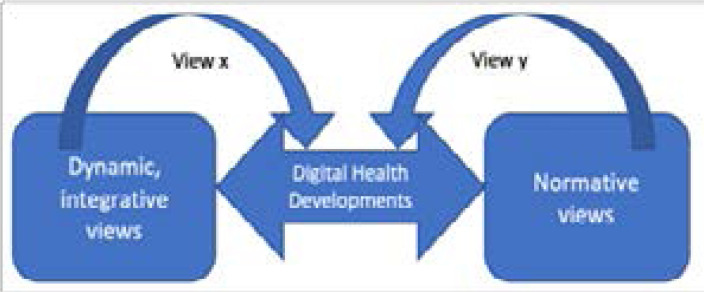
Multiple views of digital health developments (source: author)

Differences in viewpoints (see figure 1) are the result of a clash of paradigms^128^, the dominant language used in framing ‘problems’^139^, and ongoing forms of orientalism, imperialism and colonialism^140^. The elucidation of the subalternised voices can bring about strong reactions, ranging from total dismissal due to complete ignorance of the situation on the ground, through to applause for bringing the issue to the fore. As an interlocutor from the US responded in a personal communication in June 2020, after reflecting on the transcript of the research interviews:

I suspect that many would have the same reaction that I have when I read about these things and when I reflect on my own experiences. And that reaction is frustration from a sense of impotence to do anything about the situation.

He continued:

*I have seen the attitudes of the leaders with power... in their interactions withan African country (as well as the reciprocal attitudes ofthe African country's leaders), and even when these people are well-intentioned, they simply perpetuate the types of attitudes... that have governed the relationships between Africa and the rest of the world for literally centuries*.

And concluded that:

*Africa must simply take charge of its destiny, without the ‘help’ from the outside that is so tempting, since it comes with the promise of money, and also without the blessing from the outside, since the blessing always comes with strings attached*.

In the execution of their national health strategies, many African countries rely heavily on funding from external, non-African partners. With the acceptance of external funding, the architecture and functionalities of digital health platforms – and the expropriation of data – are influenced by organisations from outside the continent. This influence is invariably enmeshed with institutionl, societal, and philosophical Eurocentrism, which masks the variety of practices and cultures, the complexity and specificities of local contexts, and the agency of local and national actors in African countries^151^.

*Richard Scott and Maurice Mars, from their research on South Africa, argue that “a sustainable eHealth solution is best designed and developed organically and interactively with stakeholders within the context and setting in which it will be applied, and in alignment with the existing health, education, and technology enterprises*”^162^. This call for engagement is echoed in the words of Philip Alston, special rapporteur on extreme poverty and human rights for the United Nations: “*... in order to reduce the harm caused by incorrect assumptions and mistaken design choices, digital welfare systems should be co-designed by their intended users and evaluated in a participatory manner*”^173^.

The quotes from Africa come from embedded witnesses of local agency and local demand. They are insightful, critical, and evidence-based. They point to the fact tat datafication and the introduction of digital platforms for digital health, and the numerous associated apps, raise pertinent questions, and even suspicions, in relation to their security and use, including, for instance, how to view issues of data and technological sovereignty^184^, ^195^. Ministries of health in many African countries operate nationwide general electronic health information platforms, as well as some specific care related platforms. These platforms often emerged from (relatively well-funded) HIV-related patient-level care and internationally developed health information systems that facilitate reporting, often geared towards satisfying donor demands. National systems are expanding, for instance, with specific care related platforms like Laboratory Information Management System reporting on viral load testing 2016. Ideally, such platforms should reside computer systems located within the premises of national governments.

In addition, ministries of health are involved in various experiments and projects, like the development and piloting of electronic health record (EHR) systems. Other developments include applications for e-partograph, the piloting of telehealth, systems for the notification of maternal deaths, the implementation of blended learning, and the monitoring of clinical mentoring. Unfortunately, most literature on digital health developments in Africa reflect institutional, societal, and philosophical views set in European schemes, which can subalternise local and national assessments of evidence. Many universities located in Europe or the US operate technical assistance services from departments of global health ‘for the South’. In African countries, these Euramerican services are often fronted by local companies, or preferred by officials influenced by an innovator's dilemma^21^, corruption, donor-dependencies, or eurocentric training. Examples are the University of Washington in e-learning^22^,^17^, working through the International Training and Education Centre for Health (I-TECH) in Zimbabwe, University of Maryland for AIDS relief in Zambia, and Oslo University which supports District Health Information Software 2 (DHIS2). The list is endless. Exceptions are few and far between, but they do exist, for instance, the African COVID-19 FAIR developments (VODAN) being managed from Uganda^23^.

Cooperation in international health involves languages and views from at least two perspectives: that of African communities and that of partner environments (figure 1). Guidance for the latter is contained in national and international government policies, codicils, and bilateral and other agreements. Guidance for the former is set in a variety of local, national and other structures, and includes so-called traditional structures. However, language dominance and enshrined Eurocentric institutionalisation, social practices and philosophies on practices, especially in the sciences, obscure Southern voices^9^, ^139^.

Although literature and guidance are increasingly gravitating towards co-development as ‘the way to go’, environmental, skills and cultural differences still hamper effective co-development24. There is much to be done to remove all the barriers to cooperation. In relation to a technology lock-in currently experienced in a West African country, one respondent explained as follows:

Sometimes, people say things like ‘technology is there’. But as soon as you start scraping, they say things like ‘you cannot get the source code, this is protected’. Not because the NGO does not want to give it to you, but because the NGO has subcontracted with somebody who subcontracted with somebody else. In the end you get lost in a maze about who decides on giving you the code. We have had this experience just recently. Then in the end, they tell you, ‘it is very simple, you can do it yourself’. But, if it is very simple, give it to us! But no...

We currently have a very big project in the country, where a donor has given money, we built something, and the project is now at the end. We are in the exploitation phase. And now we discover that the stack of components that were built actually need some licences. Very strong licences. The problem is not the licences, but that without those very small stacks (that are not part of the big project) the entire infrastructure doesn't work. So, we must pay for the licences. Since we did not negotiate the price of these licence with these people from the start, now that they realise that we depend on those components, anything can happen. Prices they decide; there is no possible negotiation because theentire structure was built on these components and we are dependent on these components now. We said that thisdigital platform was given to us, that we should put many services, which we did. Now we realise we cannot live without this infrastructure, which, by the way, we do not know how it is built. We just know that it is there.

This narrative mirrors the experiences of Jabiri Bakari, CEO of the e-Government Agency of Tanzania, during the IFIP WG 9.4 conference in Dar es Salaam in May 20192518. At that time:

Bakari stressed that the developing countries should realize the power that ‘home-grown’ ICT solutions have in solving many existing challenges of these countries. He argued that the challenges that befall the developing countries are as a result of their overreliance on the ICT solutions from the developed countries. The problems of developing countries is not a complex one, thus they do not need complex ICT solution either. He expects that the policymakers, researchers, innovators, implementers, and the consumers of ICT solutions to be aware of the potentials that ‘home-grown’ ICT solutions have in solving the challenges of developing countries, by using the internally developed human capital capacities and novelties advocated by research and innovation in the ICT field.^26^,^19^

The quotes represent critical voices that are often absent in literature on digital health development. They highlight the need for:

- Ethical guidance on ownership and values of cooperation
- Involving local communities as the drivers and sustenance of development
- A reality check on envisioned needs with available infrastructures
- Sensitivity to cultural differences in interactions and respectful handling of any information, including data on the context and content of discussions and assessments There is a general lack of evidence about the benefits of imported digital health interventions in low-resource settings, or that they are more useful than so-called traditional methods. The quotes contained in this paper clearly show the need for discussions on available infrastructure to precede discussions about using infrastructure. There is a risk that overly focussing on the latest digital health application could crowd out developments based on necessity, such as basic infrastructure developments, like community networks. One interlocutor pertinently described the large number of well-funded, externally-proposed pilots – experiments – as ‘noise’ and expressed the real need ‘to reduce the noise’.

From these narratives there emanates a call for orthopraxy: ‘doing the right thing’. There is a clear need for digital health guidance that includes and values local cultures and that also allows Africans to express their views freely. During the research, Fred Mweetwa of Macha Works in rural Zambia commented that, once such guidelines are available “[it will unlock the world for the first time in global history, therefore, allowing development to flourish naturally by allowing Africans to define their own needs and work with Western friends as partners”.

## Conclusion

We live in a highly diverse world. Views on the fundamental nature of knowledge, reality, and existence – in other words, philosophies – vary. Shared concepts and categories, their properties and the relations between them – ontologies – depend on the physical and social features of the locale. This is also true in the narratives around digital health, where Eurocentrism and structures of exclusion have been droning out local voices. In digital health, such diversities and different views of reality come to a head^270^. Therefore, there is a need for more complexity, more inclusion, and more intellectual rigour that commits to listening to an array of voices. For development to be effective, these diverse views need to be reconciled – it is imperative to find common ground from conceptualisation through to practice. Digital health interventions cannot be seen from a single, Eurocentric point of view, but need to be informed by the local understanding of needs, use local resource and be cognisant of local technical possibilities.
